# Growth during the first year in infants affected by neonatal abstinence syndrome

**DOI:** 10.1186/s12887-018-1327-0

**Published:** 2018-11-05

**Authors:** Tammy E. Corr, Eric W. Schaefer, Ian M. Paul

**Affiliations:** 1 Penn State College of Medicine, Department of Pediatrics, P.O. Box 850, 500 University Drive, Hershey, PA 17033-0850 USA; 2 Penn State College of Medicine, Department of Public Health Sciences, Hershey, PA USA

**Keywords:** Neonatal abstinence syndrome, Neonatal opioid withdrawal syndrome, Infant growth, Infant nutrition, Pediatric obesity, Behavioral feeding, Comfort feeding, Parenting practices

## Abstract

**Background:**

Infants with neonatal abstinence syndrome (NAS) initially experience neurologic excitability, poor feeding, and/or hyperphagia in the setting of increased metabolic demand. Because the longitudinal effects of these early symptoms and behaviors on weight trends are unknown, we sought to contrast weight gain patterns through age 1 year for infants diagnosed with NAS with matched controls.

**Methods:**

Retrospective cohort of 70 singletons with a gestational age of ≥37 weeks and an ICD-9 or ICD-10 diagnosis of NAS made ≤7 days after birth with institutional follow-up matched to patients without NAS. Infants were matched on gestational age (±2 weeks), birth weight (±20 g), sex (exact), and insurance type (exact). Quantile regression methods were used to estimate 10th, 25th, 50th, 75th and 90th percentiles of weight over time.

**Results:**

The mean gestational age for an infant with NAS was 38.8 weeks (standard deviation [SD], 1.3). The mean birth weight was 3.141 kg (SD, 0.510). NAS patients had a median of 24 weights recorded between birth and 400 days (inter-quartile range [IQR], 16–32 weights). Patients without NAS had a median of 12 weights recorded (IQR, 10–16). Growth curves were similar over the first 400 days of life. Patients with NAS had non-significantly higher and lower estimated weights for the 90th and 10th percentiles, respectively.

**Conclusion:**

Infants with a diagnosis of NAS grew similarly to controls during their first year. Given the frequently-encountered NAS symptoms of hyperphagia and irritability, future studies may evaluate whether early differences in caregiver feeding exist and whether they have longer-term impacts on growth.

## Background

Neonatal abstinence syndrome (NAS) is a growing public health problem both nationally and globally [1–4]. Infants affected by neonatal abstinence syndrome display a number of symptoms and behaviors related to neurologic excitability including increased tone, tremors, hyperthermia, tachypnea, excessive crying, and increased time in an awake state [5]. Additionally, these infants often exhibit poor feeding with an uncoordinated suck as well as symptoms of gastrointestinal dysfunction such as regurgitation and emesis and loose or watery stools [5]. This constellation of neurologic and gastrointestinal symptoms may result in caloric intake that is inadequate and fails to meet the increased metabolic demands of the symptomatic infant resulting in hyperphagia [6].

Studies focused on caloric intake and growth of infants affected by NAS in the immediate neonatal period are inconsistent and sparse [6–9]. While some studies suggest weight loss in the neonatal period is greater in drug-exposed infants [7, 9], other studies report infants seem to compensate for this hypermetabolic state by increased intake [6], while still others propose that this hyperphagia can lead to excessive weight gain [8]. Even less is known about the feeding patterns and subsequent growth of these affected infants as they age [10].

Infants affected by NAS are symptomatically irritable and difficult to soothe. These characteristics along with early hyperphagia may lead to the development of aberrant feeding behaviors by caregivers with a tendency towards feeding to comfort. While infants have an innate ability to control their caloric intake [11, 12], parental feeding practices can alter eating behavior and affect subsequent weight gain and growth [13]. Therefore, we sought to estimate the weight patterns through age 1 year for infants affected by NAS compared with matched controls without NAS. We hypothesized that infants affected by NAS would have larger weight gains than those of matched controls and a tendency towards obesity.

## Methods

### Design

In this retrospective cohort study, electronic medical records (EMR) of all newborns (*N* = 13,718) hospitalized at the Penn State Milton S. Hershey Medical Center (HMC; Hershey, PA) between July 2008 and March 2016 were queried. HMC is a tertiary care center with a level IV neonatal intensive care unit (NICU) and an active maternal-fetal medicine (MFM) program. Data extracted from the EMR included birth weight, type of delivery (vaginal or Cesarean), sex, gestational age, singleton versus multiple birth, NICU stay, insurance status (public, private, self-pay), receipt of drugs (morphine, phenobarbital, clonidine) during birth hospitalization, total number of inpatient and outpatient visits within 13 months after birth, and weights and lengths entered in the EMR during inpatient or outpatient visits. Where data were missing from searchable fields of NAS patients, they were manually extracted from the clinical chart. For all transferred newborns with a diagnosis of NAS (*N* = 127), pre-transferal data were obtained by physically reviewing outside records.

### Participants

Analysis was restricted to newborns of singleton birth with a gestational age ≥ 37 weeks and a diagnosis of NAS made ≤7 days after birth who had ≥3 weights recorded after discharge with ≥1 weight recorded between 100 and 400 days to ensure adequate follow up. NAS cases were identified using the ICD-9 diagnosis code 779.5 (drug withdrawal syndrome in a newborn) and the ICD-10 code P96.1 (neonatal withdrawal symptoms from maternal use of drugs of addiction). Each NAS patient was matched to 1 patient without NAS on gestational age (±2 weeks), birth weight (±25 g), sex, and insurance (private vs. public, exact match) using a greedy matching algorithm [14]. While the acceptable range for matching on gestational age was wide, the majority were exact matches, and only 1 match had a 2-week difference. Eligible matches were well singletons with no NICU stay, a gestational age of ≥37 weeks with record of birthweight who had ≥3 weights recorded after discharge with ≥1 weight recorded between 100 and 400 days. A total of 2900 patients without NAS met these inclusion criteria. For matching, self-pay was combined with Medicaid and missing insurance status was imputed as private.

### Data analysis

We used a quantile regression model appropriate for longitudinal data to estimate 10th, 25th, 50th (median), 75th, and 90th percentiles of weight (measured in kg) as a function of time after birth. The penalized fixed-effects model in the R package “Regression Quantiles for Panel Data (rqpd)” was used to estimate the percentile curves [15]. The model includes separate intercepts for each patient to account for the correlation among repeated weights measured for a patient. Regularization was used to estimate the intercepts with the amount of regularization controlled by a tuning parameter (λ). Separate models were fitted for patients treated with NAS and their matched controls so that λ could be specified differently in each model and adjusted accordingly for the smaller number of weights recorded for matched controls. The parameter λ was set equal to 50 for NAS patients and to 20 for matched controls.

To estimate non-linear percentile curves, we used restricted cubic splines [16] with knots at each quartile of weight and a knot at 2 days after birth. The knot at 2 days was used to model the early and expected loss of weight in the first days after birth. All weights recorded from birth to 400 days after birth were used. Three obvious errors in recorded weights were deleted (e.g. a value of 0 kg). Final percentile curves were only shown to 390 days (approximately 13 months after birth). Non-parametric bootstrapping with 1000 bootstrap samples was used to test for differences between NAS patients and matched controls for each percentile curve. In these bootstrap samples, matched pairs were randomly selected, and all weights from selected patients were used in respective models fits in each bootstrap sample.

We conducted 2 subgroup analyses. First, we compared percentile curves of patients who did and did not receive pharmacologic therapy for NAS. Second, we compared patients with NAS who received pharmacologic therapy to their matched controls. The same methods were used as above with the following changes: λ was set to 20 and only 4 total knots were used (instead of 5) for patients with NAS who did not receive pharmacologic therapy due to the smaller number of weights recorded for this subgroup.

## Results

**Fig. 1:**
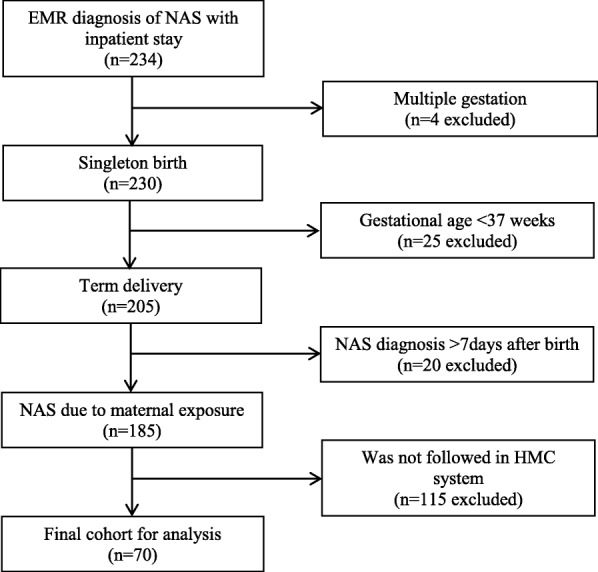
Flow diagram of patients retained for analysis

Among the 234 neonates with a diagnosis of NAS and documented inpatient stay at our center, 70 (30%) met all inclusion criteria and were included in the final analysis (Fig. 1). Thirty-seven percent of the NAS population was early term, 50% term, and 13% post term. NAS patients were primarily insured by Medicaid (81%). Similar to controls, about one third of NAS infants were born via C-section, and the majority (89%) were inborn. Nearly 75% of NAS patients had a NICU admission. Median lengths of stay were 11.1 days for patients with NAS and 2.2 days for controls (Table 1).

**Table 1:** Demographic and birth characteristic of infants with NAS^a^ and matched controls

Variable	NAS	Matched Controls	
	(*n* = 70)	(*n* = 70)	
Sex^b^			
Male	30 (43%)	30 (42.9%)	
Female	40 (57%)	40 (57.1%)	
Gestational age (weeks)^b^			
37	13 (19%)	13 (19%)	
38	13 (19%)	12 (17%)	
39	25 (36%)	28 (40%)	
40	10 (14%)	10 (14%)	
41	9 (13%)	7 (10%)	
Birth weight (kg)^b^			
Median	3.030	3.033	
(Interquartile range)	2.746–3.465	2.750–3.465	
Insurance^b^			
Private	13 (19%)	13 (19%)	
Medicaid/self-pay	57 (81%)	57 (81%)	
			*P*-value
Transferred from outside hospital	8 (11%)	0 (0%)	0.003
Type of delivery			0.990
Vaginal	48 (69%)	47 (67%)	
Cesarean	22 (31%)	23 (33%)	
NICU^c^ stay	52 (74.3%)	0 (0%)	N/A^d^
Total length of stay (days)			< 0.001
Median (Interquartile range)	11.1 (5.3–22.3)	2.2 (1.9–2.6)	

*NAS*^a^, Neonatal Abstinence Syndrome; ^*b*^matched characteristic; *NICU*^*c*^, Neonatal Intensive Care Unit; *N/A*^*d*^*,*not applicable as controls were required to have no NICU stay; thus, the groups are different by definition

Fifty (71%) of the NAS patients were < 1 day old at the time of NAS diagnosis. An additional 14% were diagnosed on the day following birth, and the remaining 14% of patients were diagnosed between ages 2–5 days. Thirty-six (51%) of NAS patients did not receive pharmacologic treatment. Among NAS patients who required pharmacologic therapy, nearly all (97%) received treatment with morphine with 4 patients (12%) requiring the addition of a second agent (phenobarbital or clonidine) to manage their symptoms. One patient received treatment with phenobarbital alone.

Patients with NAS had a total of 2072 weights recorded between birth and 400 days of age (median = 24, inter-quartile range 16–32) compared to 974 weights recorded (median = 12, inter-quartile range 10–16) during the same time period for matched controls. The majority (61%) of the weights for patients with NAS were recorded during the initial birth hospitalization with a median of 12 weights recorded during the birth hospitalization and a median of 9 weights recorded between newborn discharge and 400 days. In contrast, for matched controls, the median number of weights recorded during the birth hospitalization was 3, and a median of 10 weights were recorded between newborn discharge and 400 days.

**Fig. 2:**
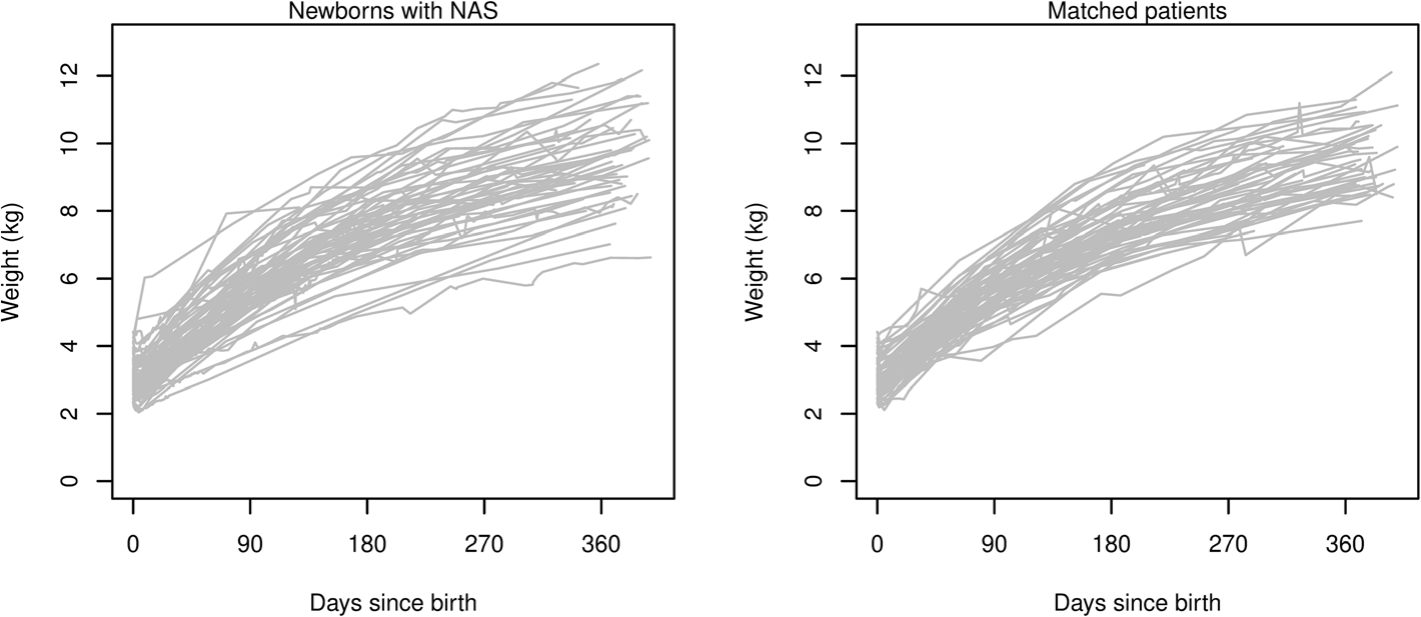
Individual growth trajectories of weight for patients with neonatal abstinence syndrome (left) and matched comparison patients (right) show similar growth patterns

**Fig. 3:**
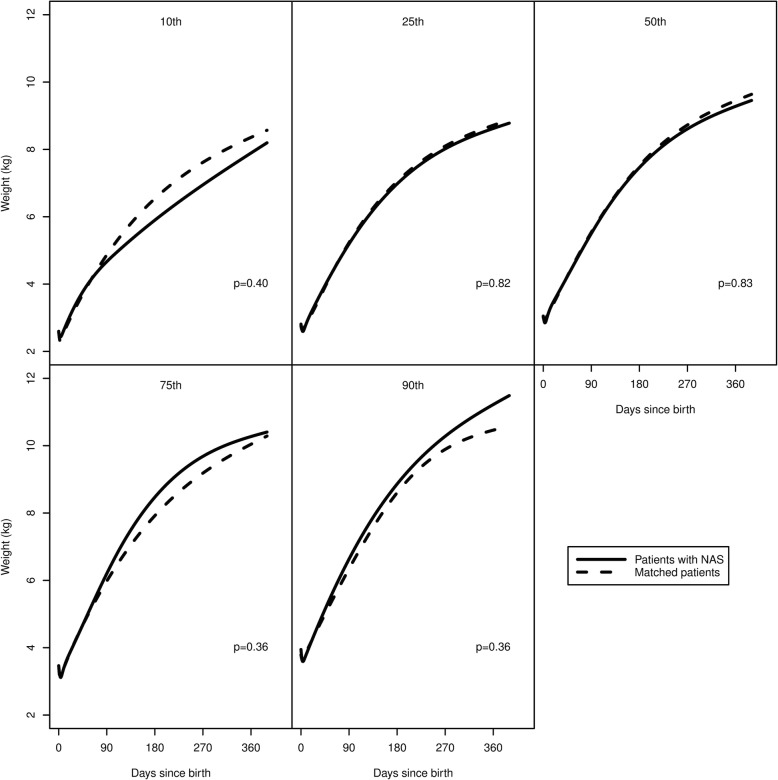
Estimated percentile curves for patients with neonatal abstinence syndrome (NAS) and their matched comparison patients show similar growth patterns between patients with and without NAS

Figure 2 displays individual growth trajectories of patients with NAS and their matched counterparts, while Fig. 3 shows the estimated percentile curves of weight as a function of time after birth for NAS patients and matched controls. Percentile estimates were generally similar between groups, although the 10th and 90th percentiles were wider for NAS patients. However, no differences were statistically significant between groups for any percentile.

**Fig. 4:**
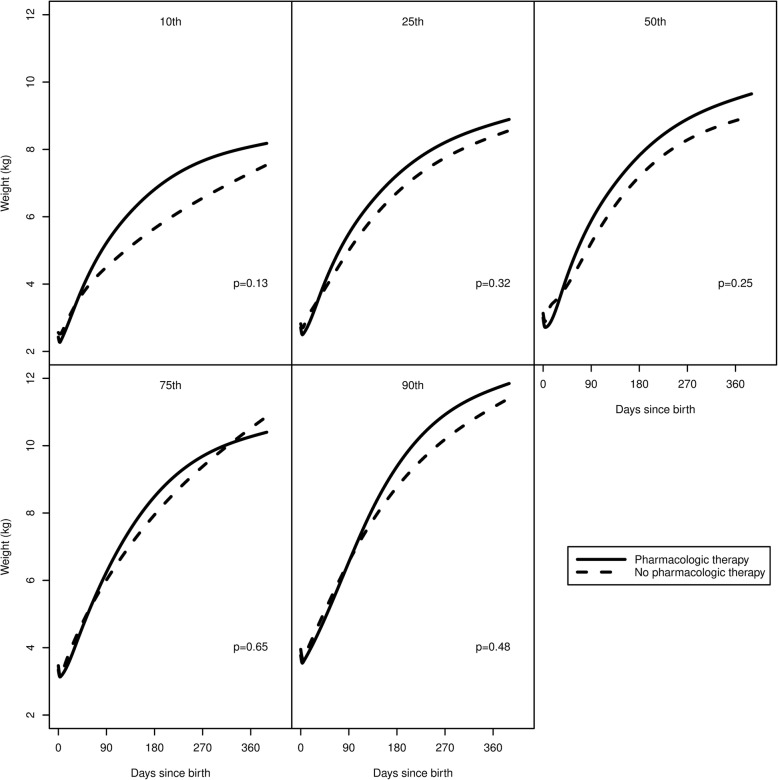
Estimated percentile curves for patients with neonatal abstinence syndrome (NAS) stratified by pharmacologic therapy reveals no difference in growth between infants receiving pharmacologic treatment and those who do not

**Fig. 5:**
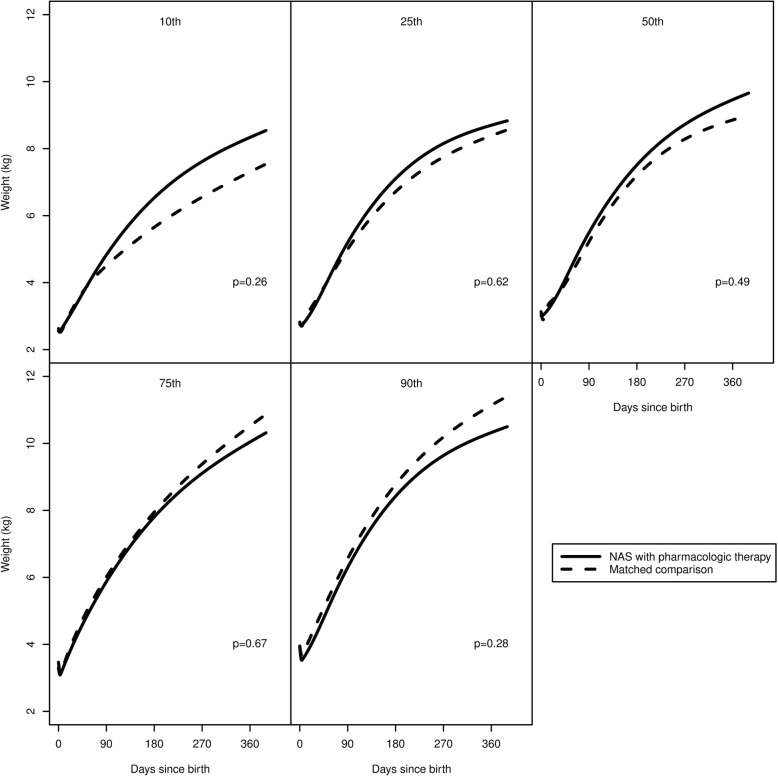
Estimated percentile curves for patients with neonatal abstinence syndrome (NAS) who received pharmacologic therapy and matched comparison patients demonstrate no significant difference in growth over the first year

In a subgroup analysis, Fig. 4 shows the percentile curves of patients with a diagnosis of NAS who received pharmacologic therapy (*N* = 34) and those who did not (*N* = 36). Differences between groups were non-significant for each percentile. In a separate subgroup analysis, Fig. 5 shows percentile curves of patients with NAS requiring pharmacologic therapy and matched controls. Differences were again non-significant.

## Discussion

This retrospective, pilot analysis of data from a single center failed to reveal significant growth differences between birth and 1 year among those infants diagnosed with NAS when compared with matched controls. Further subgroup analysis of those NAS infants pharmacologically treated compared to matched controls did not demonstrate growth differences. These results conflict with our a priori hypotheses, which reflected known feeding difficulties and hyperphagia among infants with NAS.

Pediatric growth is a complex, multifactorial process influenced by genes, nutritional intake, the environment, overall health, and socioeconomic status (SES). In the newborn period, the NAS population is unique in its nutritional needs. The hypermetabolic state resulting from symptoms of withdrawal in combination with poor feeding places this patient population at risk for excessive weight loss in the neonatal period [7, 9]. While the neonate may compensate for this hypermetabolic state by increased intake [6], there is some evidence that these eating habits may lead to undue weight gain [8]. Our study failed to support either of these patterns of growth.

Instead, consistent with a previous investigation by Vance et al. [10], we found similar weight gain trends between infants affected by NAS and their matched counterparts. This likeness existed when comparing all infants with a diagnosis of NAS to matched controls and when comparing controls only to NAS patients with more severe disease who were treated with pharmacologic therapy. Reasons for this lack of difference may be due to our small sample size of just 70 patients. Indeed, there appears to be a trend, albeit non-significant, towards NAS patients having higher estimated weight values for the 90th percentile and smaller estimated values for the 10th percentile.

It is reasonable to presume differences may exist in the growth of this population for a number of reasons. Similar to previous study findings [3, 17], NAS patients cared for at our center were predominantly insured by Medicaid, a proxy for lower socioeconomic status [18]. There is an abundance of data that suggest children affected by poverty are at risk for abnormal weight gain. Wright et al. revealed that children of deprivation were 2.2 times more likely than children with adequate resources to have failure to thrive [19], and more recent data from developing countries suggest children from low-income households are at risk for both undernutrition and overnutrition [20, 21]. In developed countries such as the United States, there are numerous studies that indicate there is an inverse relationship between weight and SES [22–24].

However, the burden of NAS is experienced by members of all socioeconomic statuses, and deprivation alone is not the only reason to suspect variance in growth. There is compelling evidence to suggest early feeding behaviors affect childhood eating habits and weight [25, 26]. Hyperphagia and significant irritability are characteristic symptoms in newborns affected by NAS. In an effort to soothe these agitated infants, caregivers may feed to comfort under the incorrect assumption the infant is crying secondary to hunger. While infants have an innate ability to control their caloric intake [11, 12], parental feeding practices can alter eating behavior and affect subsequent weight gain and growth [13]. Therefore, it is reasonable to suspect that this population is at risk for development of abnormal feeding behaviors with a consequent tendency towards obesity. It is also equally plausible to presume this behavior is modifiable as recently demonstrated in the INSIGHT trial with infants not affected by NAS [27].

There are a number of limitations to our study. Our data are retrospective and gathered from a single center with a fairly homogenous population. Many patients ultimately received their post-discharge care outside the HMC system, and the resulting sample size is small and may conceal actual differences that exist in growth of this vulnerable population. Additionally, utilizing a hospital database for research depends on correct ICD coding. Failing to assign the relevant diagnostic code for an infant who displays symptoms of NAS may lead to a falsely-low appreciation of the true extent of this syndrome at our institution. Conversely, inappropriately assigning a diagnosis of NAS to an infant being observed for NAS may lead to improper selection of the desired study population. Indeed, in our dataset, over half of the patients with a diagnosis of NAS did not receive pharmacologic management, suggesting that their symptoms were mild or they were inaccurately assigned such a diagnosis. Future research using a larger database with access to long-term follow-up data may clarify whether differences in growth exist between patients affected by NAS and their non-affected counterparts.

## Conclusion

Infants with a diagnosis of NAS grew similarly to matched controls in this small, retrospective sample from a single center. Future studies may evaluate whether early differences in caregiver feeding exist, and if so, whether they have longer-term impacts on growth of these infants.

## Abbreviations

EMR: Electronic medical records
HMC: Hershey Medical Center
IQR: Inter-quartile range
MFM: Maternal fetal medicine
NAS: Neonatal abstinence syndrome
NICU: Neonatal intensive care unit
SD: Standard deviation
SES: Socioeconomic status
